# Unveiling the Axial Hydroxyl Ligand on Fe—N_4_—C Electrocatalysts and Its Impact on the pH‐Dependent Oxygen Reduction Activities and Poisoning Kinetics

**DOI:** 10.1002/advs.202000176

**Published:** 2020-04-27

**Authors:** Xin Yang, Dongsheng Xia, Yongqiang Kang, Hongda Du, Feiyu Kang, Lin Gan, Jia Li

**Affiliations:** ^1^ Shenzhen Geim Graphene Center Tsinghua Shenzhen International Graduate School Tsinghua University Shenzhen 518055 P. R. China; ^2^ Laboratory for Computational Materials Engineering Tsinghua Shenzhen International Graduate School Tsinghua University Shenzhen 518055 P. R. China

**Keywords:** density functional calculations, Fe—N_4_—C catalysts, fuel cells, oxygen reduction, poisoning kinetics

## Abstract

Fe—N—C materials have shown a promising nonprecious oxygen reduction reaction (ORR) electrocatalyst yet their active site structure remains elusive. Several previous works suggest the existence of a mysterious axial ligand on the Fe center, which, however, is still unclarified. In this study, the mysterious axial ligand is identified as a hydroxyl ligand on the Fe centers and selectively promotes the ORR activities depending on different Fe—N_4_—C configurations, on which the adsorption free energy of the hydroxyl ligand also differs greatly. The selective formation of hydroxyl ligand on specific Fe—N—C configurations can resolve contradictories between previous theoretical and experimental results regarding the ORR activities and associated active configurations of Fe—N—C catalysts. It also explains the pH‐dependent ORR activities and, moreover, a previously unreported pH‐dependent poisoning kinetics of the Fe—N—C catalysts.

## Graphical abstract

An axial hydroxyl ligand on Fe—N—C catalysts under oxygen reduction reaction (ORR) is revealed by using density functional theory calculations combined with electrochemical experiments. This uncovered ligand can close the gap between recent theoretical and experimental results regarding the active sites of Fe—N—C catalysts. It also leads to pH‐dependent ORR activities and pH‐dependent poisoning by thiocyanates.

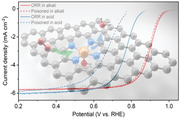

Single atomic Fe coordinated with four planar nitrogen atoms embedded in carbon materials (Fe—N—C)^[^ ^1^, ^2^, ^3^ ^]^ represent one of the most promising nonprecious electrocatalysts for oxygen reduction reaction (ORR) in fuel cells due to their low cost and high catalytic activity. However, the active site nature of the Fe—N—C catalysts remains elusive. Experimental studies based on X‐ray adsorption spectroscopy and Mössbauer spectroscopy suggested that the catalytic active site of Fe—N—C catalysts consisted of single atomic Fe center coordinated with four nitrogen atoms (namely, Fe–N_4_ moieties),^[^ ^4^, ^5^, ^6^, ^7^, ^8^, ^9^, ^10^, ^11^, ^12^ ^]^ including different planar Fe—N_4_—C configurations (Figure S1, Supporting Information) such as bulk Fe—N_4_—C_10_ (generally denoted as D1),^[^ ^10^, ^12^ ^]^ Fe—N_4_—C_12_ (D2),^[^ ^9^, ^11^, ^12^ ^]^ Fe—N_4_—C_8_ (D3),^[^ ^11^, ^12^ ^]^ and two other edge Fe—N_4_—C structures (armchair and zigzag, denoted as ZZ‐edge and AM‐edge, respectively). It was suggested that D1 and D3 configurations were most active.^[^ ^4^, ^5^, ^8^, ^12^ ^]^ However, theoretical calculations using the planar Fe—N_4_—C structures predicted comparable or even inferior activity of Fe–N_4_ moieties compared to conventional metal‐free C/N catalysts,^[^ ^13^, ^14^, ^15^, ^16^, ^17^ ^]^ contrary with the experimental finding that the active Fe center plays an important role in ORR electrocatalysis. Recently, several experimental studies^[^ ^9^, ^12^ ^]^ evidenced a nonplanar Fe—N—C configuration in as‐prepared materials induced by a fifth axial ligand exist (e.g., N) on the Fe center in D3, whereas no fifth ligand existed on D1 and D2. This fifth ligand likely contributed to the high ORR activity of Fe—N—C catalysts. And some other studies also emphasized the synergetic effect of Fe‐containing species and Fe–N_4_ moieties which can considerably boost the ORR activity.^[^ ^18^, ^19^ ^]^ Nevertheless, theoretical studies proposed that the fifth axial ligand might be identified to be an OH ligand on the Fe center of D1 or ZZ‐edge configurations,^[^ ^20^, ^21^, ^22^, ^23^ ^]^ which was spontaneously formed as an ORR intermediate at one side of Fe center and significantly enhanced the ORR activity on the other side.^[^ ^23^ ^]^ As a result, the nature and the origin of this axial ligand and the resulted ORR‐relevant Fe—N—C configurations are still controversial and to be clarified.

Besides the unclarified active structural configurations, previous studies also reported pH‐dependent activities of the Fe—N—C catalysts, showing higher catalytic activities in electrolytes with higher pH values.^[^ ^6^, ^24^, ^25^, ^26^, ^27^, ^28^, ^29^, ^30^, ^31^ ^]^ The origin of this pH dependency also remains unclear and controversial. Some researchers ascribed the relatively higher activity in alkaline solution to the existence of Fe/Fe_3_C phases which were catalytically active in alkaline but not in acidic solution,^[^ ^24^, ^31^ ^]^ while others suggested that the decreased ORR in acid was due to the protonation of N‐groups which coexisted in Fe—N—C catalysts.^[^ ^27^, ^28^, ^29^, ^30^ ^]^ None of the above studies provide insight into the intrinsic pH‐dependency of the Fe—N—C active site.

In this study, we reveal that a fifth ligand does exist on the Fe—N—C catalyst and can be identified as a hydroxyl ligand, which forms under specific electrode potentials on selected Fe—N_4_—C configurations (D1, D3, and edge sites). The adsorption free energy of the hydroxyl ligand on the Fe center is dependent on the Fe—N_4_—C configuration as well as the pH value of the electrolyte (more favorably in alkaline condition). This hydroxyl ligand substantially decreases the bonding strength of ORR intermediates on Fe center and therefore accounts for the high catalytic activity of Fe–N_4_ moieties. It also leads to the pH‐dependent activity of Fe—N—C catalysts and a previously unreported pH‐dependent poison kinetics, which cannot be explained by the nitrogen‐axial‐ligand model. The revealed ORR‐relevant structural models based on density functional theory (DFT) calculations combined with electrochemical experiments close the gap between recent experimental and theoretical results.

We constructed five types of Fe—N_4_—C structures (Figures S1 and S2, Supporting Information), denoted as D1, D2, D3, AM‐edge, and ZZ‐edge, respectively. The free energy diagrams for ORR on these structures were then calculated (methods for free energy diagram calculations are described in detail in the Supporting Information). **Figure** **1**a presents the free energy diagram for ORR on D1, showing that when the overall reaction potential increased to 0.53 V, the last elementary step (proton/electron transfer to *OH) reached equilibrium; thus, the OH ligand would stay at the Fe center, becoming a potential‐limiting step (PLS) and corresponding to an overpotential of 0.70 V. For comparison, free energy diagrams of ORR on other Fe—N_4_—C structures are also shown in Figure S3 (Supporting Information). Notably, except for D2 structure whose PLS is the transition from *O to *OH, the PLS for all other Fe—N_4_—C structures is also the last step. These results demonstrate that the catalytic ORR activity is mainly limited by the desorption of *OH (except D2), which binds on the Fe center over strongly. Figure 1b compares the ORR activity (represented by the overpotential *η*_ORR_) of the five Fe—N_4_—C structures, suggesting decreasing ORR activity in the order of D2 > AM‐edge > D1 > ZZ‐edge > D3. However, this is in contrary with previous experimental results that the D1/D3 structure is mainly responsible for ORR activity while D2 is largely inactive.^[^ ^4^, ^8^, ^12^ ^]^ In addition, the free energy diagram of ORR on a metal‐free C/N site^[^ ^32^ ^]^ is also shown in Figure S4 (Supporting Information), and the calculated overpotential (0.56 V) is much smaller than that of D1/D3 structure, contradictory with previous experimental findings that the Fe–N_4_ catalyst was much more active than the metal‐free C/N sites.^[^ ^4^, ^5^, ^6^, ^7^, ^8^, ^9^, ^10^, ^11^, ^12^ ^]^

**Figure 1:**
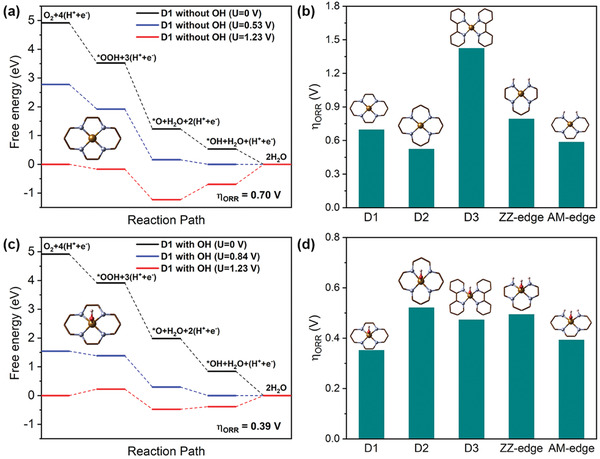
a) Free energy diagram for oxygen reduction reaction (ORR) on bulk‐hosted Fe–N_4_ structure (D1). b) Computational ORR overpotentials for different Fe—N_4_—C structures. c) Free energy diagram for ORR on bulk‐hosted Fe–N_4_ structure with an axial OH ligand. d) Computational ORR overpotentials for different Fe—N_4_—C structures with an axial OH ligand.

Considering the overstrong binding of *OH on the Fe center, we then propose that one side of Fe centers would be ligated by OH (denoted as Fe–N_4_(OH)–C) at ORR potentials, with the other side serving as the active site for the subsequent ORR. We found that the adsorption free energy of OH ligands on different Fe—N—C configurations differs significantly (Figure S3 and Table S1, Supporting Information), being exothermic on D3 whereas most difficult for D2. The free energy diagrams and the ORR activities are then re‐evaluated and shown in Figure 1c,d and Figure S5 (Supporting Information), respectively. Significantly, for D1, the presence of OH ligand substantially enhances the ORR activity, resulting in an overpotential of 0.39 V even lower than that of Pt catalyst.^[^ ^33^, ^34^ ^]^ Except for D2, all other Fe—N—C structures have improved ORR activities with the introduction of OH ligand. The sequence of the ORR activities now changed to be D1 > AM‐edge > D3 > ZZ‐edge > D2 (Figure 1d), which is in good accordance with experimental findings.^[^ ^4^, ^8^, ^12^ ^]^ The activity of D1 was also much higher than the metal‐free C/N site (with a overpotential of 0.56 V). These results imply that the Fe–N_4_(OH)–C could be the realistic active site structure for experimentally identified ORR relevant D1 and D3, and edge‐hosted Fe–N_4_ structures.^[^ ^10^, ^11^, ^12^, ^22^ ^]^

Further DFT calculations were performed to investigate the origin of enhanced ORR activities of the Fe—N_4_—C catalyst with OH ligand. Since the transition of *O to *OH has the second highest energy barrier, the ORR activities of five Fe—N_4_—C structures with an axial O ligand (denoted as Fe–N_4_(O)–C) were also evaluated. The adsorption free energies of *OOH, *O, and *OH (ΔG_*OOH_, ΔG_*O_, and ΔG_*OH_) on Fe—N_4_—C, Fe–N_4_(O)–C, and Fe–N_4_(OH)–C structures were summarized in Table S1 (Supporting Information). The relationships between ΔG_*OOH_, ΔG_*O_, and ΔG_*OH_ are linear (**Figure** **2**a).^[^ ^13^, ^34^, ^35^ ^]^ Thus, the ORR overpotential could be exclusively evaluated by ΔG_*OH_, showing a volcano plot (Figure 2b). Without any ligand, the Fe center of the bare Fe—N_4_—C structure binds *OH too strongly (the left side of the volcano plot), while it binds *OH too weak with an axial O ligand (the right side). The optimum ΔG_*OH_ on the Fe center with an OH ligand is responsible for the reported high ORR activities. Detailed DFT analyses (see Experimental Section S6 and Figures S6 and S7 in the Supporting Information) show that, the weakened *OH adsorption on the Fe–N_4_(OH)–C structure compared to the bare Fe—N_4_—C structure is due to decreased proportion of *σ* bonding states (normally strong) and increased *π* bonding states (normally weak) between the d orbitals of the Fe center and the *p* orbitals of oxygen intermediates.

**Figure 2:**
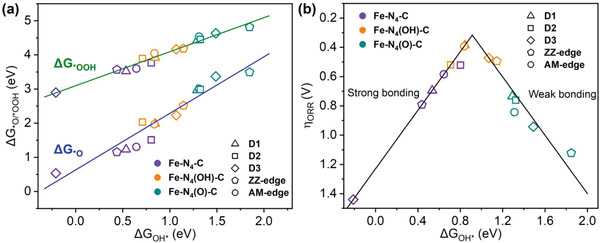
a) Scaling relationships between the adsorption free energies of *OH (ΔG_*OH_) and *O (ΔG_*O_) (green line) or *OOH (ΔG_*OOH_) (blue line) for the five Fe—N_4_—C structures with different axial ligands. b) Volcano plot between ΔG_*OH_ and the ORR overpotential for five Fe—N_4_—C structures with different axial ligands.

In the following part, we conducted several designed electrochemical measurements on a home‐made single atomic Fe—N—C catalyst to provide experimental evidences for the presence of the axial hydroxyl ligand on the Fe–N_4_ sites (**Figure** **3**a, for more details see the Supporting Information). This Fe—N—C catalyst was prepared by using Fe‐doped metal‐organic framework as the precursor following a previously reported method,^[^ ^36^ ^]^ and mainly consist of atomic Fe–N_4_ moieties. As shown in Figure S8 (Supporting Information), no crystalline phase was observed in the X‐ray diffraction pattern, indicating the absence of Fe nanoparticles. Aberration‐corrected scanning transmission electron microscopy images directly show the exclusive formation of single Fe atoms. The Mössbauer spectrum also indicates the dominate formation of the well‐known D1 Fe–N_4_ site,^[^ ^5^, ^9^, ^11^, ^12^ ^]^ without the formation of Fe nanoparticles. All these results demonstrate that the active sites of the as‐prepared Fe—N—C catalyst are mainly D1 Fe–N_4_ moieties.

**Figure 3:**
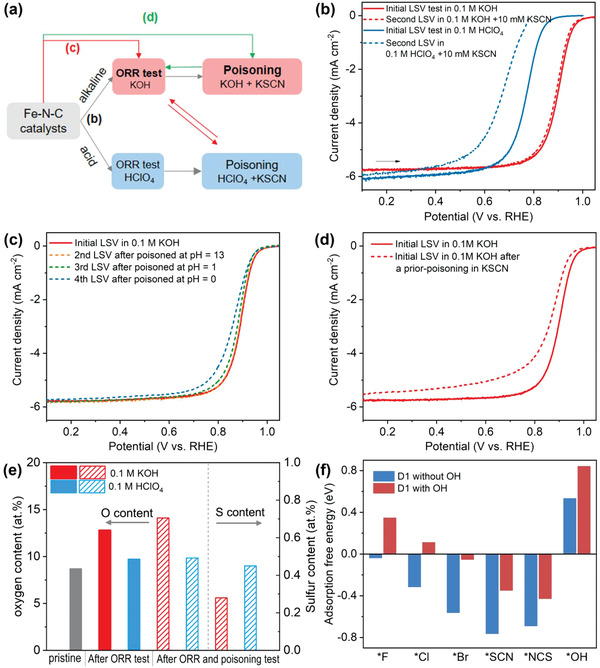
a) Illustration of designed ORR and poisoning experiments on the Fe—N—C catalysts. b) ORR LSV curves of the Fe—N—C catalyst initially measured in O_2_‐saturated 0.1 m HClO_4_ or 0.1 m KOH and second measured after adding 10 × 10^−3^ m KSCN in the same electrolyte. c) ORR LSV curves of the Fe—N—C catalyst initially measured in O_2_‐saturated 0.1 m KOH, then remeasured in 0.1 m KOH after poisoning at pH 13 (0.1 m KOH + 10 × 10^−3^ m KSCN), pH 1 (0.1 m HClO_4_ + 10 × 10^−3^ m KSCN) and pH 0 (1 m HClO_4_ + 10 × 10^−3^ m KSCN) for 10 s. d) Comparison of the ORR LSV curves initially measured in O_2_‐saturated 0.1 m KOH and after a direct prior poisoning in KSCN solution. e) XPS analysis of the oxygen and sulfur contents after the ORR and poisoning experiments as described in (b). f) DFT‐calculated adsorption free energies of *OH, *SCN, *NCS, *F, *Cl, and *Br on D1 structure with and without an axial OH ligand.

The first electrochemical experiment is the evaluation of pH‐dependent ORR activities of the Fe—N—C catalyst (Figure 3b, following testing procedure (b) in Figure 3a). Significantly higher ORR activity in alkaline solution than acidic is unveiled. This can be rationalized by the fact that, in addition to the effect of electrode potential, the axial OH ligand could also form more easily on the Fe center in alkaline solution than in acidic due to much higher concentration of OH^−^,^[^ ^37^ ^]^ leading to more catalytically active Fe–N_4_(OH)–C structure and thus higher activity in alkaline solution. In contrast, the observed pH‐dependent ORR activity cannot be explained by the previously proposed N ligand on the Fe center.^[^ ^9^, ^12^ ^]^ Cyclic voltammetry (Figure S9, Supporting Information) further shows different Fe redox behaviors (Fe^II^ + OH^−^ ↔ Fe^III^(OH) − e^−^) in N_2_‐saturated acidic and alkaline condition,^[^ ^38^ ^]^ with an obvious Fe redox peak at ≈ 0.75 V in acid but a broad peak below 0.6 V in alkaline. This indicates the formation of OH ligand on the Fe center is more favorable in alkaline compared to acidic condition, even without ORR.

The second evidence for the OH ligand comes from previously unreported pH‐dependent poisoning kinetics of the Fe—N—C catalysts. It has been reported that anions like SCN^−^, F^−^, Cl^−^, and Br^−^ would poison the Fe active center, and such poison effect has been applied as an important method to judge the existence of the Fe‐N_4_ active site.^[^ ^39^, ^40^, ^41^, ^42^ ^]^ However, little attention has been paid to the pH‐dependence of the poisoning effect. Herein, we conduct the poisoning measurements in both acidic and alkaline electrolytes (Figure 3a), during which an initial ORR measurement by linear scanning voltammetry (LSV) was performed followed by adding 10 × 10^−3^ m KSCN and subsequently a second ORR measurement. While the ORR activity was greatly depressed in acid, the activity changed little in alkaline solution or decreased very slowly even if being poisoned for a longer time (Figure S10, Supporting Information). We hypothesize that the much weaker poison effect in alkaline solution could be ascribed to the competitive adsorption of hydroxyl and SCN^−^ at the Fe center. After the initial ORR measurement in alkaline solution, large amounts of hydroxyl ligands could form on the Fe centers and thus hinder further adsorption of SCN^−^.

To verify this hypothesis, we further conducted designed poisoning experiments in 0.1 m KOH. In Figure 3c (following testing procedure (c) in Figure 3a), the ORR activity was first measured in 0.1 m KOH and therefore OH ligands formed on the Fe centers, then the catalyst was transferred and immersed in 10 × 10^−3^ m KSCN with different pH values (13, 1, and 0). Due to much lowered concentration of OH^−^, immersing in the KSCN solution at a lower pH value results in the replacement of OH ligand by SCN^−^, thus the Fe site could be poisoned. As expected, the ORR activity was well preserved after poisoned at pH = 13 but obviously decreased after poisoned at pH = 0. Figure 3d (following testing procedure (d) in Figure 3a) presents another experiment result where the Fe–N_4_ sites are directly poisoned without an initial ORR measurement to avoid potential‐induced adsorption of OH ligand, thus the SCN^−^ could poison the Fe center. Under this condition, the catalyst does exhibit obvious activity loss. Therefore, all the designed poisoning experiments support our hypothesis that there exists the competitive adsorption of hydroxyl and SCN^−^ on the Fe center in alkaline solution, which accounts for the observed pH‐dependent poisoning kinetics.

X‐ray photon spectroscopy (XPS) was further conducted to probe the different adsorption behaviors of hydroxyl and SCN^−^ after the electrochemical measurements. As shown in Figure 3e, the oxygen content in the Fe—N—C catalyst after the ORR measurement in KOH was substantially higher (12.8 at%) compared to that in the pristine catalyst (8.7 at%). In contrast, the oxygen content in the catalyst increased little after the ORR test in acid solution (9.6 at%). Increased oxygen contents due to carbon oxidation can be ruled out as the potential was set below 1.0 V/RHE and only one potential scan was applied during the ORR measurement, neither it can be ascribed to residual KOH as no K signal was detected by XPS (Figure S11, Supporting Information). Instead, the increased oxygen content after ORR measurement strongly supports the adsorption of oxygen‐containing groups (OH) on the Fe centers particularly under the alkaline condition. The pH‐dependent poisoning effect is also corroborated by a higher atomic ratio of sulfur (0.45 at%) adsorbed on the catalyst after being poisoned in the acid solution compared to that after being poisoned in the alkaline (0.28 at% S). These results clearly suggest that the hydroxyl group adsorbed on the Fe centers after the ORR measurement in alkaline solutions hinders the Fe‐N_4_ active sites from being poisoned by adsorption of SCN^−^.

The effect of hydroxyl ligand on the poisoning kinetics was further corroborated by DFT calculations (Figure 3f; Table S2, Supporting Information). Without the hydroxyl ligand, our comparative calculations indicate that SCN^−^ anion would bind to Fe center with S atom, forming an axial *SCN adsorbate. The *SCN shows a highly negative adsorption energy on the Fe center, thus blocking the activity site and suppressing the ORR activity.^[^ ^40^ ^]^ However, with an OH ligand, the adsorption free energy of *SCN is significantly increased by about 0.4 eV, leading to a weaker poisoning effect. Likewise, an OH ligand on the one side of Fe center weakens the adsorption of *OH on the other side. Although the adsorption free energy of *SCN on the Fe–N_4_(OH)–C is still lower than that of *OH, the largely different concentration of SCN^−^ and OH^−^ in alkaline solution may considerably reduce the gap between the adsorption stability of *SCN and *OH,^[^ ^37^ ^]^ resulting in the suppression of SCN^−^ poisoning in alkaline solution. It could be expected that halogen anions would also exhibit pH‐dependent poisoning kinetics due to the largely different adsorption free energies on the Fe centers with/without OH ligand, as also confirmed by the DFT results (Figure 3f).

In conclusion, by using DFT calculations combined with electrochemical experiments, we reveal the formation of an axial hydroxyl ligand on the Fe center of specific Fe—N_4_—C configurations (D1, D3, and edge Fe–N_4_ sites). This exotic OH ligand can be formed under certain electrode potentials as an intermediate during ORR electrocatalysis or directly from the alkaline solution (as schematically summarized in **Figure** **4**). The OH ligand substantially weakened the following adsorption of ORR intermediates on the other side of the Fe center, accounting for the high ORR activities of the experimentally reported Fe—N_4_—C configurations. The more easily formed hydroxyl ligand in alkaline solution contributes to the pH‐dependent ORR activity of the Fe—N_4_—C catalyst. Moreover, the competitive adsorption between the hydroxyl ligand and poisoning anions results in a previously unreported pH‐dependent poisoning kinetics of the Fe—N_4_—C catalyst. The revealed active site structures offer important insights into the electrocatalytic activities of Fe—N—C materials, bridging previously unresolved gaps between theoretical and experimental findings.

**Figure 4:**
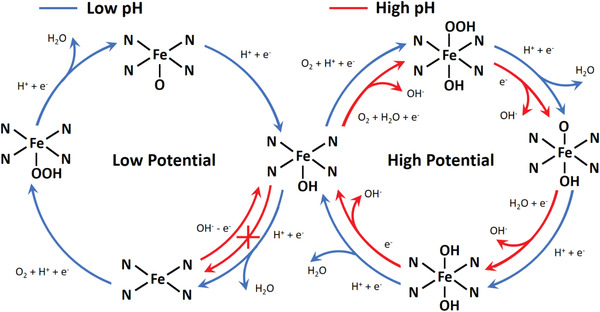
Schematic illusion of reaction pathways for ORR on Fe—N_4_ moieties in acidic and alkaline solutions under ORR working potentials.

## Conflict of Interest

The authors declare no conflict of interest.

## Supporting information

Supporting Information
