# Supplementary material for: Unveiling the Axial Hydroxyl Ligand on Fe—N4—C Electrocatalysts and Its Impact on the pH‐Dependent Oxygen Reduction Activities and Poisoning Kinetics
Source: Adv Sci (Weinh). 2020 Apr 27;7(12):2000176. doi: 10.1002/advs.202000176 (PMC7312417; doi:10.1002/advs.202000176)
Supplement: Supplementary file 1 — Supporting Information [file ADVS-7-2000176-s001.pdf]

Copyright WILEY-VCH Verlag GmbH & Co. KGaA, 69469 Weinheim, Germany, 2018.

Supporting Information

Unveiling the axial hydroxyl ligand on Fe-N_4-C electrocatalysts and its impact on the pH-dependent oxygen reduction activities and poison kinetics

Xin Yang, Dongsheng Xia, Yongqiang Kang, Hongda Du, Feiyu Kang, Lin Gan, and Jia Li**

Experimental Section

Calculational details**:** All spin-polarized calculations were performed within the framework of density functional theory (DFT), as implemented in Vienna *ab initi*o Simulation Package (VASP) [1]. The projected augmented wave (PAW) potential and generalized gradient approximation of Perdew-Burke-Ernzerhof (PBE) functional were employed to describe the electron-ion interaction and exchange-correlation energy, respectively.[2] The energy cutoff for the plane wave expansion was set to 400 eV. The vacuum distance normal to the sheet was larger than 15 Å to eliminate the spurious interaction between the periodically repeated images. All atoms were relaxed by using conjugate gradient algorithm until the force on each atom was less than 0.01 eV/Å. The Brillouin zones of bulk-hosted, AM-interstice and ZZ-interstice Fe-N_4-C structures were sampled by Γ-centered 3 × 3 × 1 *k*-points, and the Brillouin zones of AM-edge and ZZ-edge structures Fe-N_4-C structures were sampled by Γ-centered 3 × 1 × 1 *k*-points. The D3 functional was employed to describe the nonnegligible van der Waals interaction in our system.[3] An implicit solvent scheme was adopted as implemented in VASPsol to simulate the real solution environment.[4]

Material synthesis**:** The Fe-N-C catalyst used in this work was obtained by carbonizing a Fe-doped zinc-containing zeolitic imidazolate frameworks (Fe-ZIF-8) precursor at 900 ℃ for 3h in Ar. The detailed synthesis method of the Fe-ZIF-8 precursor was depicted as follows. Specifically, 2-methylimidazole (657 mg, 8 mmol) was dissolved in 15 ml methanol in beaker I. $Zn(NO_3)_2\cdot 6H_2O$ (605 mg, 2 mmol) and $Fe(acac)_3$ (70 mg, 0.2 mmol) were dissolved in 30 ml methanol in beaker II. Then, beaker II was added into beaker I with vigorous stirring for 1h at room temperature. The mixing solution then suffered from solvothermal reaction at 120 °C for 4h. The obtained product was washed with DMF (3 times) and methanol (2 times) and finally dried at 60 ℃ in a vacuum oven for over 12 h.

Physical characterizations**:** The powder X-ray diffraction pattern of the as-prepared Fe-N-C catalyst was recorded on a LYNXEYE XE-T machine using Cu K_α X-ray with a scan rate of 5° min^{-1}. A X-ray photoelectron spectroscopy test was conducted, using Al K_α X-ray source, on a PHI 5000 VersaProbe Ⅱ (Thermo Fisher) equipment to analyze the chemical states of the doping N within the surface-near region of the Fe-N-C catalyst. An ^{57}Fe Mössbauer spectrum of the catalyst was measured at room temperature with a source of ^{57}Co. The velocity calibration was performed with an α-Fe foil. TEM imaging and selected area electron diffraction were performed on a 300 kV FEI Tecnai G2 F30 TEM equipped with a Gatan Image Filter 965 ER system. The aberration-corrected atomic-resolution STEM image was obtained on a Thermo Fischer Themis G2 60e300 transmission electron microscope operated at 300 kV.

***Electrochemical measurements*:** All electrochemical characterizations were implemented on a potentiostat (BioLogic Science Instruments) coupled with a rotating disk electrode (RDE) system (Pine Research Instrumentation, USA) unless otherwise specified. To prepare catalyst working electrode, the catalyst powder was ultrasonically dispersed in an ethanol-water (V/V=1/1) mixing solution containing suspended Nafion® ionomer for 30 minutes, where the content of Nafion® ionomer is about 10 wt%. A Pt wire was used as the counter electrode in a standard three-electrode system. Hg/HgO/1M KOH(aq) (MMO) and Hg/Hg_2SO_4/saturated K_2SO_4(aq) (MMS) electrodes were used as the reference electrodes for 0.1 M KOH and 0.1 M $HClO_4$ electrolyte, respectively. Both MMO and MMS electrodes were calibrated to reversible hydrogen electrode (RHE), which was created by continuously bubbling pure H_2 gas onto Pt electrode surface in the appointed electrolyte. Oxygen reduction reaction (ORR) activities were measured by linear scanning voltammetry (LSV) on a glassy carbon (5 mm in diameter) rotation disk electrode (RDE) in O_2-saturated 0.1 M KOH/0.1 M $HClO_4$ with a positive scan rate of 5 mV s^{-1}. The catalyst loading was 0.4 mg cm^{-2}, and the electrode rotation speed was set at 1600 rpm. All currents were normalized by the disk area (0.196 cm^2) and corrected by non-faradic currents obtained in N_2-saturated solution. All potentials were also corrected by *iR* compensation.

Three designed poisoning experiments were conducted as described below:

Poisoning experiment 1 (Figure 3b): The ORR activity of the Fe-N-C catalyst film was first measured by LSV in 0.1 M KOH, then 10 mM KSCN was added into the electrolyte, and a second LSV was subsequently recorded. The same test procedures were also conducted in 0.1 M $HClO_4$ using a new catalyst film. The poisoning time of the catalyst film in both electrolytes was set long enough (above 30 min) to guarantee the poisoning effect.

Poisoning experiment 2 (Figure 3c): The ORR activity of the Fe-N-C catalyst film was first measured by LSV in O_2-saturated 0.1 M KOH. Then the catalyst film was immersed into 0.1 M KOH containing 10 mM KSCN (pH = 13) for 10 s, and subsequently transferred into O_2-saturated 0.1 M KOH solution for a second LSV test. Again, the tested film was re-immersed in 0.1 M $HClO_4$ solution (pH = 1) containing 10 mM KSCN for 10s, and subsequently tested in O_2-saturated 0.1 M KOH for the third LSV test. Finally, the same catalyst film was immersed into 1 M $HClO_4$ solution (pH = 0) containing 10 mM KSCN for 10s, and subsequently tested in O_2-saturated 0.1 M KOH for the fourth LSV test.

Poisoning experiment 3 (Figure 3d): To completely avoid the effect of the adsorbed OH on the SCN^--poison kinetics, direct poisoning of the catalyst ink by SCN^- was carried out before a LSV test. Typically, 2 M KSCN solution (10 ul) was first added into the catalyst ink (200 ul) for 10-min poisoning, then the poisoned ink was pipetted on RDE to perform a LSV test (solid green line).

X-ray photon spectroscopy of samples after electrochemical measurements: To prepare the XPS samples of the Fe-N-C catalysts after ORR and poisoning tests, the catalyst electrode were prepared without using Nafion ionomer. The ORR tests were performed by only one scan of linear scanning voltammetry from 0.06 to 1.0 V/RHE. The samples after the electrochemical measurement were carefully washed by ultrapure water for several times to avoid contaminations by the electrolyte and then completely dried.

***Free energy diagrams calculation*:** The extensive application of DFT calculations in electrocatalysis could be ascribed to the success of the computational hydrogen electrode (CHE) model, developed by Nørskov et al[5]. In such a theoretical framework, ORR activity of a given atomic model could be determined by simply calculating the adsorption free energies of key ORR intermediates. For ORR on $Fe-N_4$ catalysts in acidic solutions, many previous studies[6] have suggested a four-electron transfer pathway with four sub-steps which could be expressed as follows:

$O_2 + H^+ + e^- + * \rightarrow *OOH$ (1)

$*OOH + H^+ + e^- \rightarrow *O + H_2O$ (2)

$*O + H^+ + e^- \rightarrow *OH$ (3)

$*OH + H^+ + e^- \rightarrow H_2O$ (4)

Where * denotes the active site and *A indicates the adsorbate bound to the active site. The free energies of these terms and molecules in the equations could be directly derived via DFT calculations. The adsorption structures of these intermediates on D1 were given in Figure S2. The free energies of a proton-electron pair equal to their chemical potentials, which are calculated as follows:

$$u(H^+ + e^-) = u(H_2) + eU_{SHE} + k_BT \ln 10\ pH \quad (5)$$

Here U_{SHE} stands for the onset potential referenced to standard hydrogen electrode (SHE). When at a potential of 0 V versus SHE and a pH of 0, the chemical potential of a proton-electron pair could be simply represented as the free energies of a hydrogen molecule. Under such a reaction mechanism, the reaction free energies of each step are calculated as the difference between the Gibbs free energies of products and reactants:

$$\Delta G = \Delta E + \Delta ZPE - T\Delta S \quad (6)$$

Where E represents the internal energies derived from DFT calculations. ZPE is the zero-point energy, based on the Heisenberg uncertainty principle that the position and the momentum could not be determined simultaneously even at 0 K, which could be calculated by:

$$ZPE = 1/2\Sigma\ hv_i \quad (7)$$

Where h is the Planck constant, v_i are the vibrational frequencies derived from DFT calculations. TS is entropy correction at T = 298.15 K. And the entropy could be divided into three parts: translational, rotational and vibrational entropies. Only vibrational entropies were considered for periodic structures, whereas only translational and rotational entropies were considered for molecules. The translational and rotational entropies were taken from experimental values.[12] And the vibrational entropy was obtained from the calculated vibrational frequencies via the following equation[7]:

$$TS = \sum_i k_B T \ln \left[1 - e^{\left(-\frac{hv}{k_B T}\right)}\right] \quad (8)$$

Where k_B denotes the Boltzmann constant, T is the temperature. Since the difference between the zero-point energies of adsorbates on different models is really small, only the vibrational frequencies of the adsorbates on D1 structure were calculated. The computed vibrational frequencies, the derived ZPE and TS were summarized in Table S3.

For ORR proceeds in alkaline media, the reaction pathway could be presented as follows:

$O_2 + H_2O + e^- \rightarrow *OOH + OH^-$ (9)

$*OOH + e^- \rightarrow *O + OH^-$ (10)

$*O + H_2O + e^- \rightarrow *OH + OH^-$ (11)

$*OH + e^- \rightarrow OH^-$ (12)

In this case, the chemical potential of hydroxyl ion is regarded as the energy difference between a gas phase water molecule and a proton-electron pair. And the influence of pH value could also be eliminated by replacing the reference electrode SHE to reversible hydrogen electrode (RHE):

$U_{RHE} = U_{SHE} + k_BT \ln 10 \, pH/e$ (13)

Therefore, theoretical evaluation of ORR activity is independent with the pH value of electrolyte. For an ideal catalyst, the reaction free energies of each step in the four-electron transfer reaction path should be equal to the equilibrium potential of water splitting, 1.23 V versus reversible hydrogen electrode (RHE).

***Adsorption free energies of poison anions*:** Inspired by the CHE model in the calculation of free energy diagram, we computed the free energies of poison anions as the difference between the free energies of corresponding gas phase hydride molecules and hydrogen molecule. In this case, the calculation of exact free energies of poison anions was avoid. The free energies of poison anion X^- could be expressed as:

$G(X^-) = G(HX) – 0.5G(H_2)$ (14)

And the adsorption free energies of poison anions were computed as:

$\Delta G_{ads}(X^-) = G(*X) – G(*) – G(X^-)$ (15)

The vibrational frequencies, ZPE and TS of corresponding gas phase hydride were summarized in Table S4. Although some approximations have been employed in our calculations, the obtained results could reveal the ability of these poison anions to block the Fe active sites to some extent.

***Bonding analysis*:** The underlying mechanism of remarkable improvement of ORR activities induced by OH ligand is further investigated by means of projected density of states (PDOS). Taking the square planar Fe- N_4 moieties in D1 structure as a representative, belonging to point group D_{4h}, the degenerate *d* orbitals of central Fe atom have to split into four distinct orbitals, namely the d_{xy}, degenerate d_{xz} and d_{yz}, d_z^2 and d_x^2-d_y^2 orbitals, as shown in Figure S6a. For the adsorption of ORR intermediates, according to the symmetry of orbitals, the d_{xz} and d_{yz} orbitals of Fe atom involved in forming π bond with the p_x and p_y orbitals of intermediates, while d_z^2 orbital could directly be overlapped with the p_z orbital of intermediates to form σ bond, which is typically stronger than π bond. Thus, the bonding between the intermediates and Fe center is determined by the strength of σ and π bonds together. Figure S6b shows the bonding chrematistics of Fe center in D1 structure with the single-side adsorption of *OH, indicating the σ bonding between d_z^2 orbital of Fe and *p* orbital of *OH and π bonding between the d_{xz} and d_{yz} orbitals of Fe and *p* orbital of *OH, respectively, as illustrated by the overlapped area of these orbitals. For the PDOS of Fe center in D1 structure with additional adsorption of *OH on the opposite side (Figure S6c), the proportion of σ bonding states is significantly decreased. Accordingly, the interaction between *OH and Fe center is dominated by the strength of π bonding states, which depends on the filling degree of d_{xz} and d_{yz} of Fe center and is generally weaker than σ bonding. Hence, the ORR catalytic performance of Fe-N_4 moieties with OH ligand has been improved greatly due to weakened adsorption strength of intermediates. The same behavior is found for the Fe-N_4 moieties with O ligand, as shown in Figure S7. However, the adsorption of ORR intermediates is weakened excessively for the Fe-N_4 moieties with O ligand, resulting in the unchanged ORR activities.

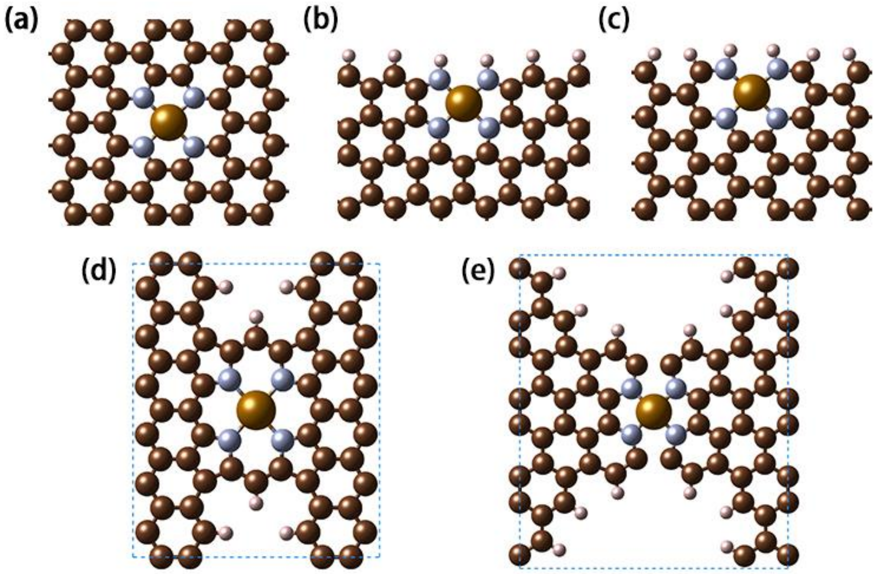

Figure S1. Atomic structures of a) D1; b) ZZ-edge Fe-N_4-C; c) AM-edge Fe-N_4-C structures; d) D2; e) D3. The yellow, light blue, brown, and white circles represent for Fe, N, C, and H atoms, respectively.

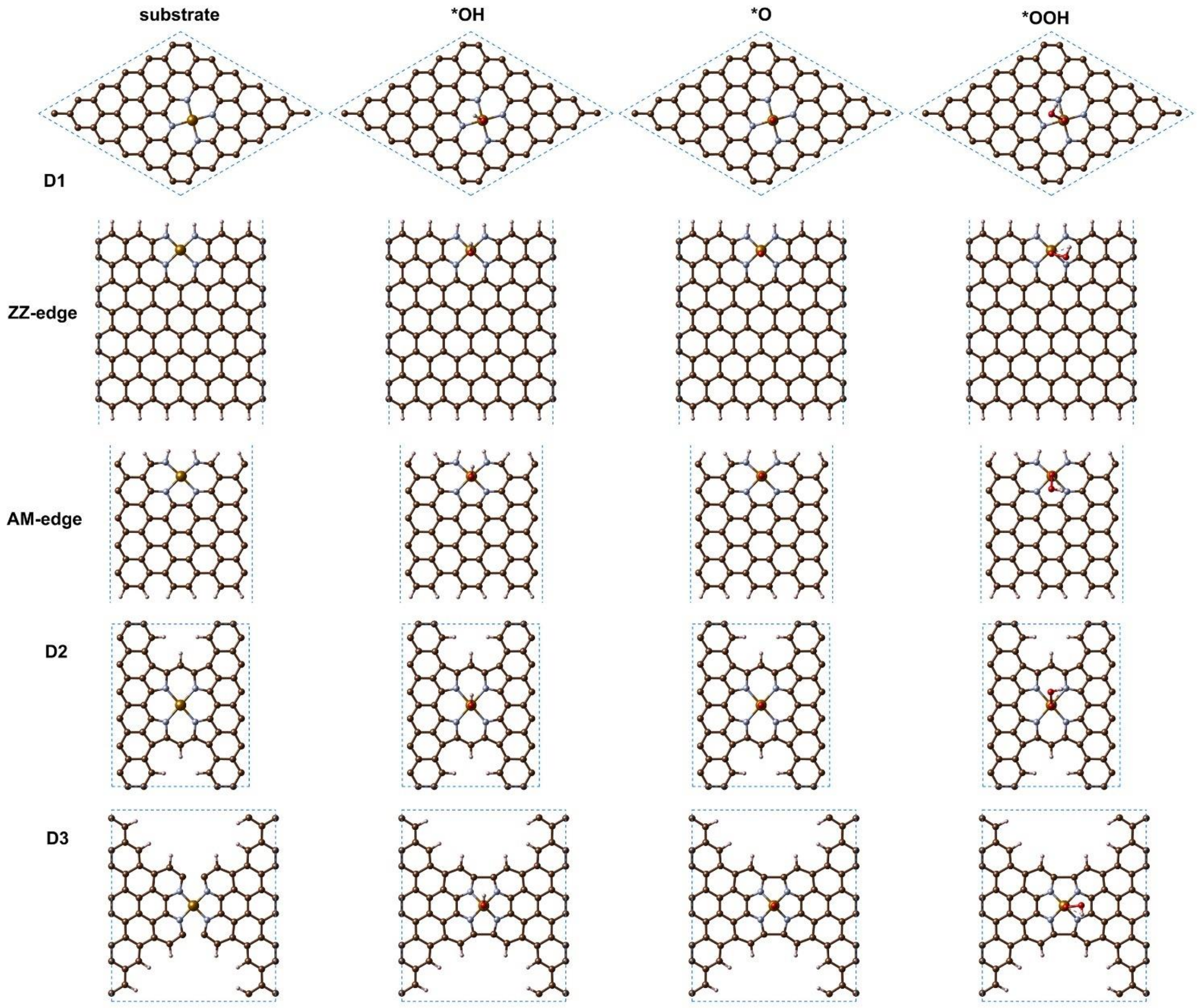

Figure S2. Atomic models of substrate, *OH, *O and *OOH of five Fe-N_4-C structures.

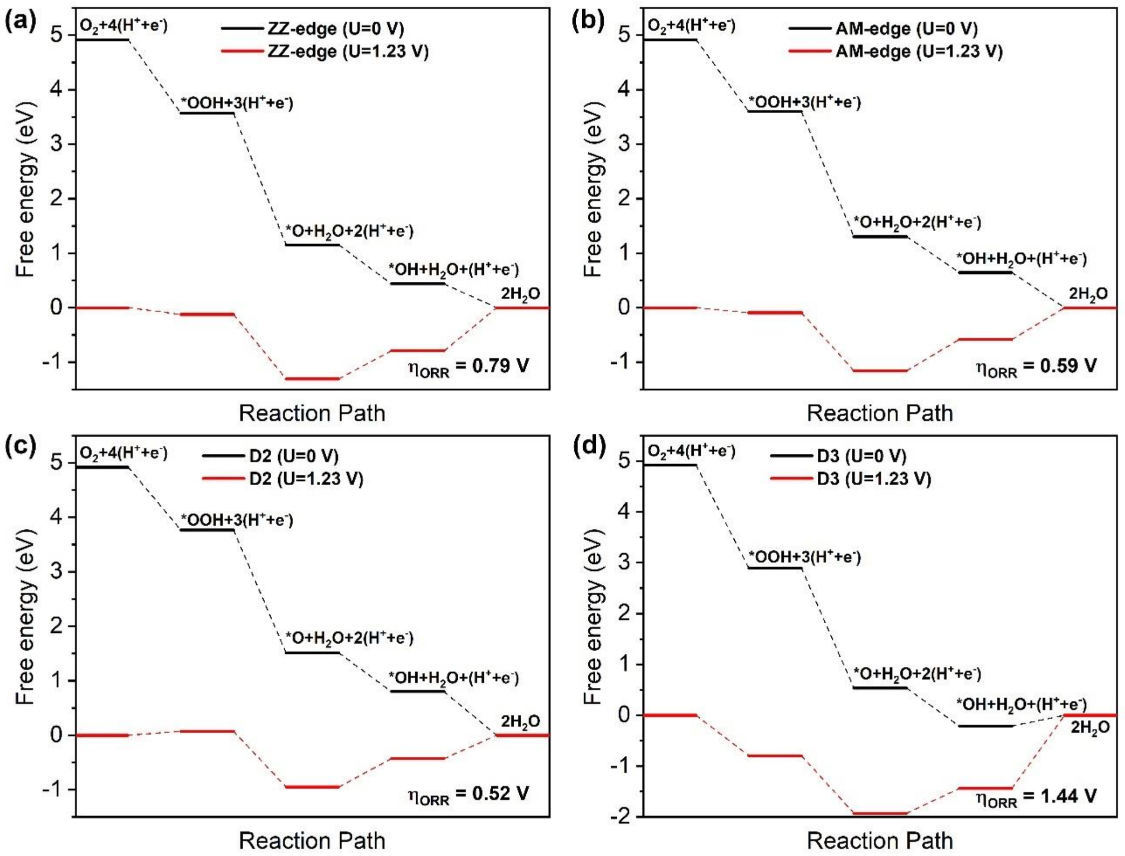

Figure S3. Free energy diagrams of ORR on a) D1; b) ZZ-edge; c) AM-edge; d) D2; e) D3 Fe-N_4-C structures.

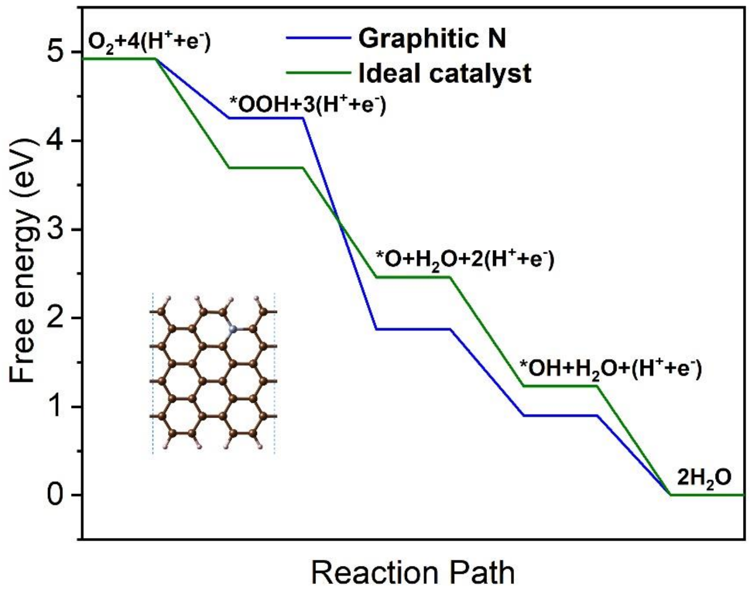

Figure S4. Free energy diagrams of ORR on ideal catalyst and the carbon atom neighboring to graphitic nitrogen. The binding energies of ORR intermediates were taken from Li *et al*'s study[8].

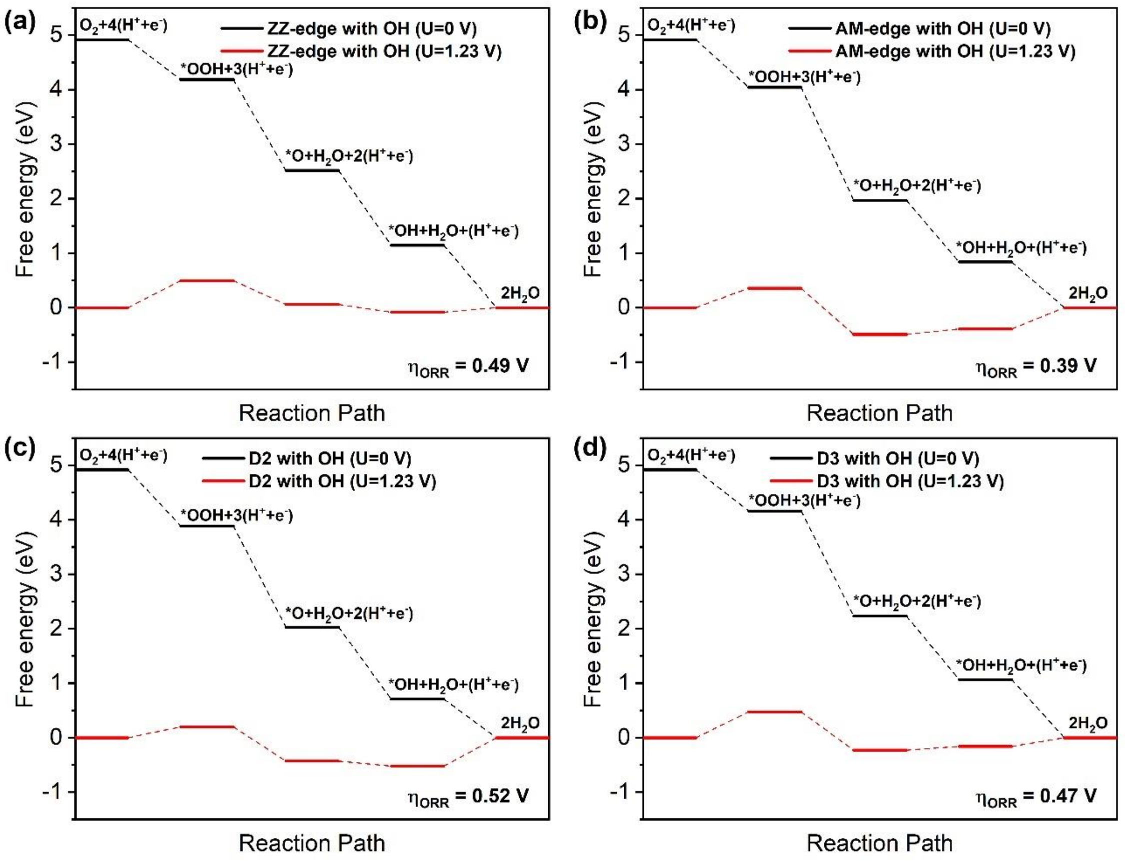

Figure S5. Free energy diagrams of ORR on a) D1; b) ZZ-edge; c) AM-edge; d) D2; e) D3 Fe-N_4(OH)-C structures.

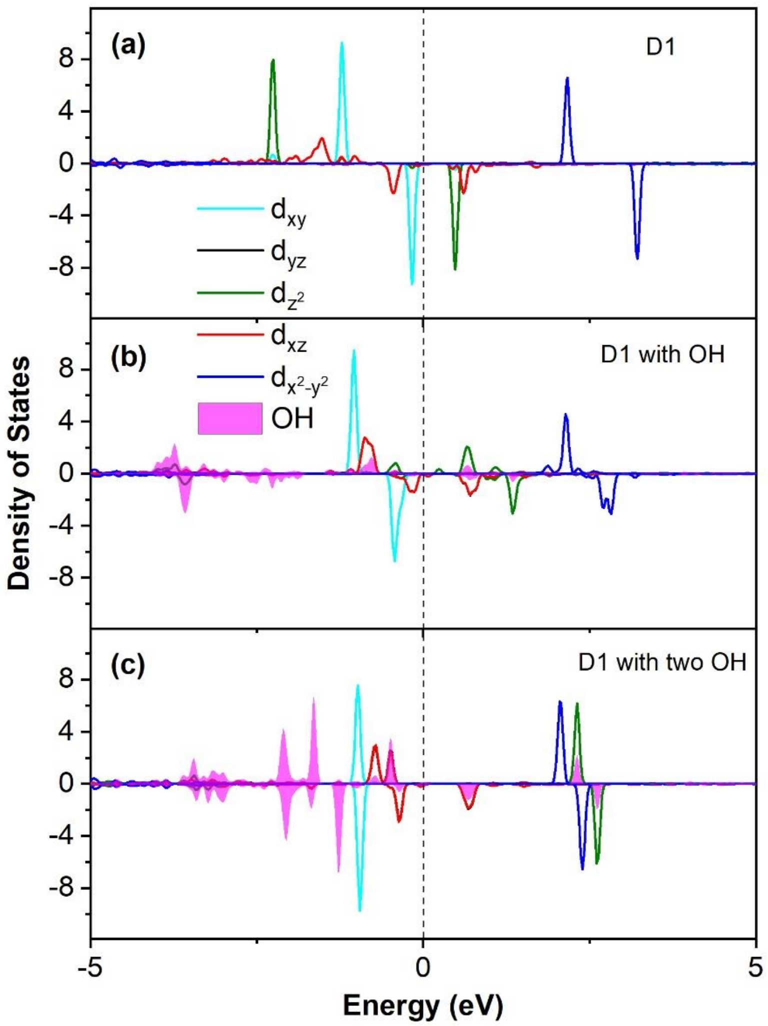

Figure S6. Projected density of states of D1 Fe-N_4-C structures with and without OH ligands.

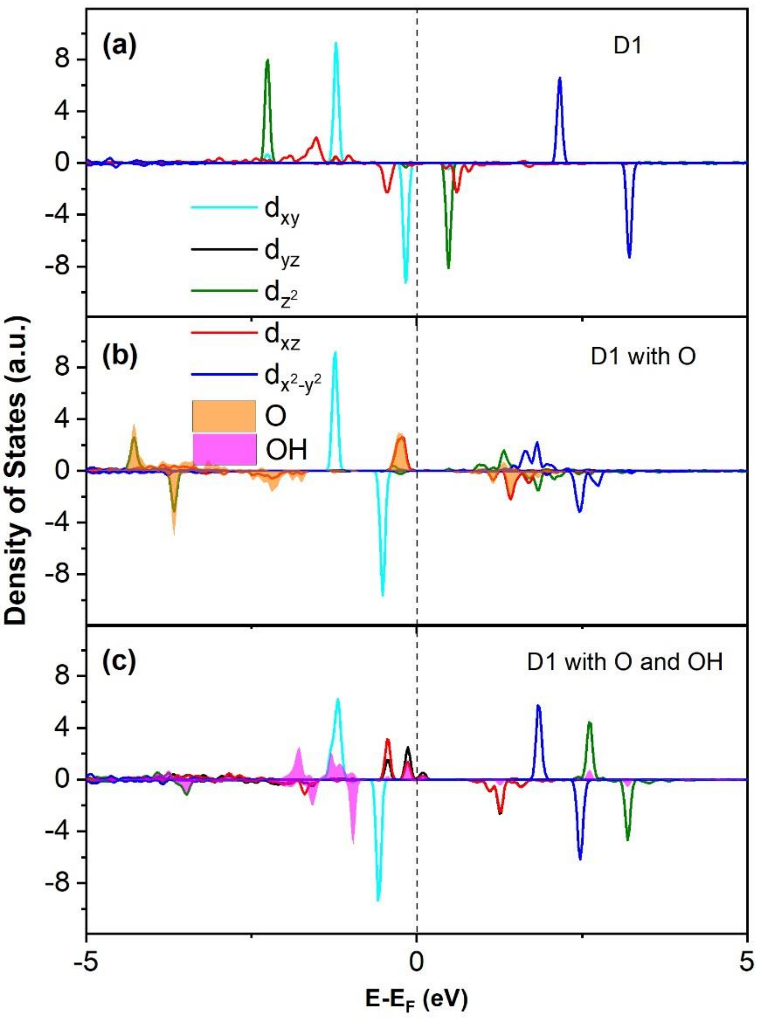

Figure S7. Projected density of states of D1 Fe-N_4-C structures with and without OH, O ligands

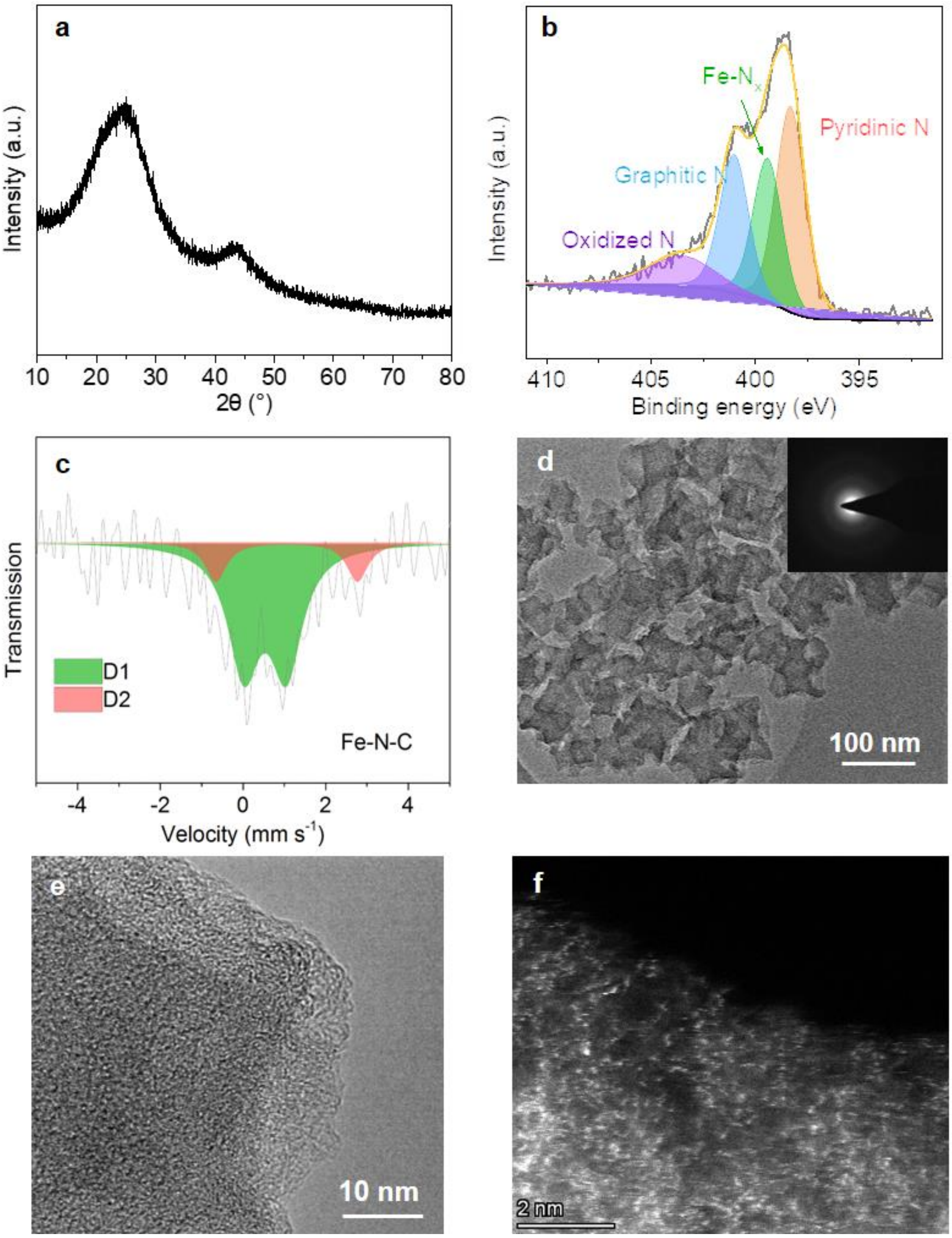

Figure S8. Physiochemical characterizations of the as-prepared Fe-N-C catalyst. (a) XRD pattern; (b) XPS of N 1s with deconvolution; (c) Mossbauer spectrum; (d) TEM image with an inset of SAED pattern; (e) High-resolution TEM image; (f) High-resolution aberration-corrected STEM image.

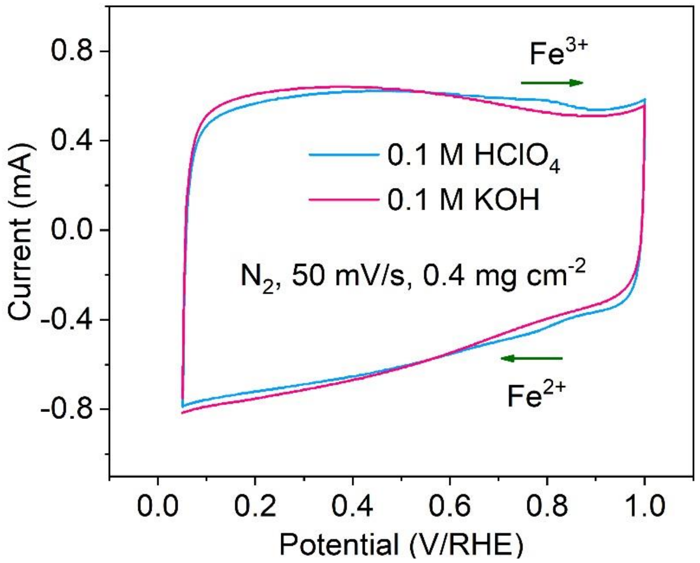

Figure S9. Cyclic voltammetry tests for the as-synthesized Fe-N-C catalysts.

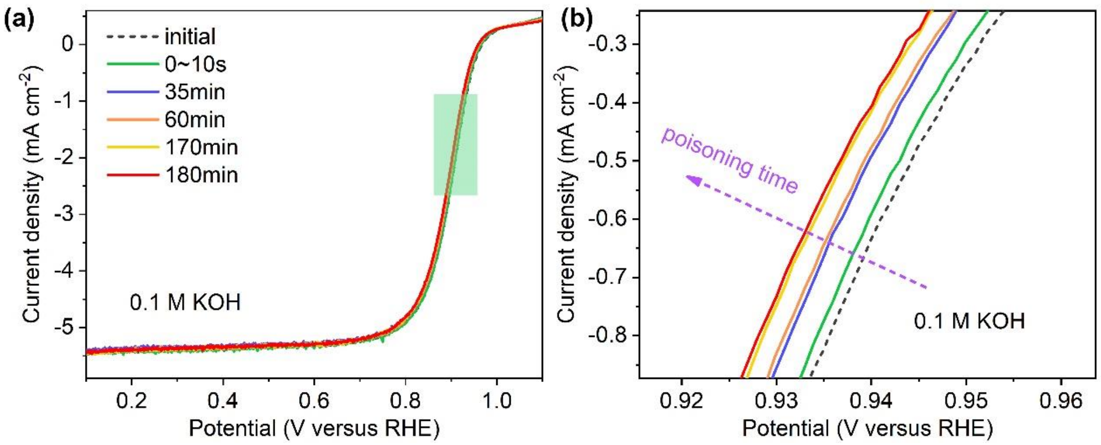

Figure S10. a) ORR polarization curves of the Fe-N_4-C catalyst after poisoned for different duration in O_2-saturated 0.1 M KOH solution; b) Magnified curves in the green domain of a).

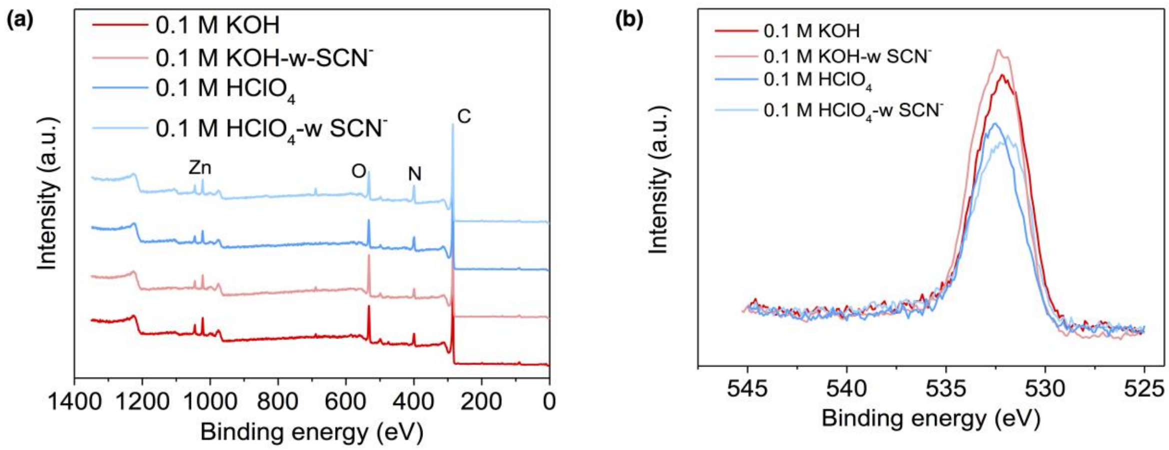

Figure S11. (a) Full XPS spectra of the Fe-N-C catalysts measured after ORR and poisoning tests under alkaline or acid conditions (following the electrochemical experiments as described in Figure 3). The spectra were normalized to the intensity of the C 1s peak at 284 eV. No K signal (377 eV for the 2s-orbital electron) can be detected. (b) O-2p narrow spectra of the Fe-N-C catalysts after the electrochemical measurements.

Table S1. Adsorption free energies of different ORR intermediates, calculated overpotential, and on-site magnetic moment of Fe center for different Fe-N-C, Fe-N(OH)-C, Fe-N(O)-C and C/N structures. The data for C/N structure were taken from Li *et al*'s study.[8]

Models	ΔG_{OOH*} [eV]	ΔG_{O*} [eV]	ΔG_{OH*} [eV]	overpotential [V]
D1	3.524	1.229	0.535	0.695
D1-OH	3.915	1.984	0.844	0.386
D1-O	4.425	2.967	1.291	0.735
ZZ-edge	3.567	1.152	0.438	0.792
ZZ-edge-OH	4.184	2.517	1.145	0.494
ZZ-edge-O	4.813	3.493	1.847	1.123
AM-edge	3.598	1.304	0.645	0.585
AM-edge-OH	4.045	1.968	0.837	0.393
AM-edge-O	4.534	3.008	1.311	0.844
D2	3.764	1.510	0.803	0.523
D2-OH	3.890	2.031	0.709	0.521
D2-O	4.451	2.983	1.324	0.761
D3	2.897	0.533	-0.210	1.440
D3-OH	4.163	2.232	1.068	0.473
D3-O	4.632	3.369	1.489	0.942
C/N	4.253	1.873	0.898	0.56

Table S2. Adsorption free energies of poison species on D1 configuration with and without OH ligand, respectively.

Poison species	ΔG_{ads} on D1 without OH ligand [eV]	ΔG_{ads} on D1 with OH ligand [eV]
*F	-0.040	0.349
*Cl	-0.318	0.114
*Br	-0.565	-0.055
*SCN	-0.767	-0.351
*NCS	-0.693	-0.431

Table S3. Calculated vibrational frequencies, ZPE and TS (T = 298.15 K) of bulk-hosted Fe-N_4 structure with different ORR intermediates.

Models	Vibrational frequencies [meV]	ZPE [eV]	TS [eV] (T = 298.15 K)
Fe-N_4-O	95.091, 22.074, 21.646	0.069	0.065
Fe-N_4-OH	109.466, 25.868, 15.812, 7.320, 4.510, 4.296	0.346	0.104
Fe-N_4-OOH	449.867, 152.305, 85.142, 62.143, 31.741, 26.870, 18.453, 12.259, 6.197	0.422	0.205
Fe-N_4-F	56.082, 14.676, 13.155	0.010	0.200
Fe-N_4-Cl	32.894, 11.665, 10.913	0.028	0.116
Fe-N_4-Br	21.035, 6.456, 3.997	0.016	0.166
Fe-N_4-SCN	263.567, 86.719, 54.689 50.230, 30.648, 14.660, 11.609, 10.474, 5.749	0.264	0.252
Fe-N_4-NCS	245.512, 108.582, 60.794, 56.561, 32.534, 17.925, 12.658, 6.343, 4.489	0.273	0.257
Fe-N_4(OH)-OH	456.901, 456.710, 105.139, 104.175, 62.495, 58.669, 36.676, 26.110, 24.219, 23.674, 21.389 9.181	0.693	0.210
Fe-N_4(OH)-F	455.310, 107.304 64.307, 57.582 26.806, 23.927, 21.643, 21.564, 17.706	0.398	0.174
Fe-N_4(OH)-Cl	454.355, 109.394, 62.955 29.620, 26.192, 24.706, 20.828, 14.601, 12.975	0.378	0.206
Fe-N_4(OH)-Br	453.926, 109.544, 62.732, 26.163, 25.579, 20.923, 18.362, 7.645, 5.060	0.365	0.257
Fe-N_4(OH)-SCN	454.694, 263.255, 109.433, 88.949, 62.558, 56.097, 52.131, 32.133, 26.225, 25.178, 20.693, 14.821, 12.880, 9.989, 4.644	0.603	0.185
Fe-N_4(OH)-NCS	455.382, 251.275, 108.963, 108.451, 63.312, 59.764, 57.907, 34.087, 25.855, 24.684, 23.106, 19.591, 17.852, 5.917, 4.361	0.630	0.342

Table S4. The vibrational frequencies, ZPE and TS (from JANAF database[9]) of molecules.

Molecules	Vibrational frequencies [meV]	ZPE [eV]	TS [eV] (*T* = 298.15 K)
H_2	533.811	0.267	0.410
H_2O	475.294, 460.463, 196.291,	0.566	0.670
HF	497.033	0.249	0.541
HCl	359.704	0.180	0.582
HBr	319.705	0.160	0.618
HSCN	322.707, 273.452, 117.558, 86.131, 48.971, 40.711,	0.445	0.628

References

[1] a) G. Kresse, J. Furthmuller, *Comput. Mater. Sci.* **1996**, *6*, 15-50; b) G. Kresse, J. Furthmuller, *Phys. Rev. B* **1996**, *54*, 11169-11186.

[2] a) J. P. Perdew, K. Burke, M. Ernzerhof, *Phys. Rev. Lett.* **1996**, *77*, 3865-3868; b) G. Kresse, D. Joubert, *Phys. Rev. B* **1999**, *59*, 1758-1775.

[3] S. Grimme, J. Antony, S. Ehrlich, H. Krieg, *J. Chem. Phys.* **2010**, *132*, 154104.

[4] K. Mathew, R. Sundararaman, K. Letchworth-Weaver, T. A. Arias, R. G. Hennig, *J. Chem. Phys.* **2014**, *140*, 084106.

[5] a) J. K. Nørskov, J. Rossmeisl, A. Logadottir, L. Lindqvist, J. R. Kitchin, T. Bligaard, H. Jónsson, *J. Phys. Chem. B* **2004**, *108*, 17886-17892; b) A. E. Russell, *Phys. Chem. Chem. Phys.* **2008**, *10*, 3607-3608; c) J. Rossmeisl, Z. W. Qu, H. Zhu, G. J. Kroes, J. K. Nørskov, *J. Electroanal. Chem.* **2007**, *607*, 83-89.

[6] J. H. Zagal, M. T. Koper, *Angew. Chem. Int. Ed. Engl.* **2016**, *55*, 14510-14521.

[7] C. Freysoldt, B. Grabowski, T. Hickel, J. Neugebauer, G. Kresse, A. Janotti, C. G. Van de Walle, *Rev. Mod. Phys.* **2014**, *86*, 253-305..

[8] M. T. Li, L. P. Zhang, Q. Xu, J. B. Niu, Z. H. Xia, *J. Catal.* **2014**, *314*, 66-72.

[9] "NIST-JANAF Thermochemical Tables", can be found under https://janaf.nist.gov/, **1998**, DOI: 10.18434/T42S31
